# Awareness of, responsiveness to and practice of patients’ rights at Uganda's national referral hospital

**DOI:** 10.4102/phcfm.v5i1.491

**Published:** 2013-06-21

**Authors:** Harriet Rachel Kagoya, Dan Kibuule, Honoré Mitonga-Kabwebwe, Elizabeth Ekirapa-Kiracho, John C. Ssempebwa

**Affiliations:** 1 School of Public Health, Makerere University, Uganda; 2 Pharmacy Department, University of Namibia, Namibia; 3 Community Medicine Department, University of Namibia, Namibia

## Abstract

**Background:**

The realisation of patients’ rights in resource-constrained and patient-burdened public health care settings in Uganda remains an obstacle towards quality health care delivery, health care-seeking behaviour and health outcomes. Although the Uganda Patients’ Charter of 2009 empowers patients to demand quality care, inequitable access and abuse remain common.

**Aim:**

The study aimed to assess level of awareness of, responsiveness to and practice of patients’ rights amongst patients and health workers (HWs) at Uganda's national referral hospital, Mulago Hospital in Kampala.

**Methods:**

A three-phase cross-sectional questionnaire-based descriptive survey was conducted amongst 211 patients, 98 HWs and 16 key informants using qualitative and quantitative data collection methods. The study was conducted in May–June 2012, 2.5 years after the launch of the Uganda Patients’ Charter.

**Results:**

At least 36.5% of patients faced a challenge regarding their rights whilst seeking health care. Most of the patients (79%) who met a challenge never attempted to demand their rights. Most patients (81.5%) and HWs (69.4%) had never heard of the Uganda Patients’ Charter. Awareness of patients’ rights was significantly higher amongst HWs (70%) than patients (40%) (*p* < 0.01). Patients’ awareness was associated with education level (χ2 = 42.4, *p* < 0.001), employment status (χ2 = 33.6, *p* < 0.001) and hospital visits (χ2 = 3.9, *p* = 0.048). For HWs it was associated with education level (χ2 = 155.6, *p* < 0.001) and length of service (χ2 = 154.5, *p* <0.001). Patients feel powerless to negotiate for their rights and fear being discriminated against based on their ability to bribe HWs with money to access care, and political, socio-economic and tribal status.

**Conclusion and recommendations:**

Awareness of, responsiveness to and practice of patients’ rights remains limited at Mulago Hospital. There is a need for urgent implementation of an integrated multilevel, multichannel, patient-centred approach that incorporates social services and addresses intrinsic patient, HW and health system factors to strengthen patients’ rights issues at the hospital.

## Abstrait

**Prise de conscience, capacité de réaction et respect des droits des patients à l'hôpital de référence national d'Ouganda**.

**Contexte:**

Le respect des droits des patients dans les établissements publics de soins de santé aux ressources limitées et surchargés de patients reste un obstacle à l'accès à des soins de santé de qualité, à la recherche de soins de santé et à la santé des patients. Bien que la Charte ougandaise des patients de 2009 permette aux patients d'exiger des soins de qualité, l'inégalité de l'accès et les abus sont monnaie courante.

**Objectif:**

Cette étude a pour objectif d’évaluer le niveau de prise de conscience, de capacité de réaction et de respect des droits des patients parmi les patients et les professionnels de la santé à l'hôpital de référence national en Ouganda, l'hôpital Mulago à Kampala.

**Méthodes:**

Une enquête descriptive sous forme d'un questionnaire transversal en trois phases a été réalisée auprès de 211 patients, 98 professionnels de santé et 16 informateurs clés en utilisant des méthodes qualitative et quantitative de collecte des données. L’étude a été réalisée en mai-juin 2012, deux et demi après l’élaboration de la Charte ougandaise des patients.

**Résultats:**

Au moins 36,5% des patients ont rencontré des difficultés quant au respect de leurs droits dans leur recherche de soins de santé. La plupart des patients (79%) ayant rencontré des difficultés n'ont jamais tenté de faire valoir leurs droits. La majorité des patients (81,5%) et des professionnels de la santé (69,4%) n'a jamais entendu parler de la Charte ougandaise des patients. La prise de conscience des droits des patients était sensiblement plus élevée chez les professionnels de la santé (70%) que chez les patients (40%) (*p* < 0.01). La prise de conscience des patients était liée au niveau d’éducation (χ2 = 42.4, *p* < 0.001), au statut professionnel (χ2 = 33.6, *p* < 0.001) et aux visites à l'hôpital (χ2 = 3.9, *p* = 0.048*)*. Pour les professionnels de la santé, elle était liée au niveau d’éducation *(*χ2 = 155.6, *p* < 0.001) et aux années de service (χ2 = 154.5, *p* < 0.001). Les patients se sentent impuissants lorsqu'il s'agit de faire valoir leurs droits et ont peur d’être victimes de discrimination quant à leur capacité à soudoyer les professionnels de santé avec de l'argent en vue de bénéficier d'un accès aux soins, et à leur statut politique, socio-économique et tribal.

**Conclusion:**

La prise de conscience, la capacité de réaction et le respect des droits des patients restent limités à l'hôpital Mulago. Il est urgent de mettre en place une approche intégrée à tous les niveaux, multicanal, centrée sur le patient, qui intègre des services sociaux et qui traite les facteurs intrinsèques liés au patient, aux professionnels de la santé et au système de santé afin de renforcer les droits des patients à l'hôpital.

## Introduction

Patients’ rights are a fundamental human right,^1, 2^ a quality assurance measure that protects patients against abuse and discrimination and promotes ethical practices. The Uganda Patients’ Charter of 2009^3^ describes a ‘set of rights, responsibilities and duties under which a person can seek and receive health care service, and empowers patients to responsibly demand for quality health care and actively participate in their care at public facilities’. Slomka^4^ showed that ‘A patient's rights centered care approach as part of the standard health care package promotes rational and ethical practices and improves health outcomes’.

Uganda is a signatory to international and regional treaties on human rights, which imply patients’ rights, including the Universal Declaration of Human rights,^1^ International Covenant on Economic, Social and Cultural Rights,^5^ African Charter on Human and Peoples’ Rights,^6^ and International Covenant on Civil and Political Rights.^7^ In Uganda's Constitution^8^ of 1995, article 287 obliges the State to uphold these treaties, and chapter 4 and article 12 recognise the World Health Organization (WHO) constitutional declaration^2^ that ‘the highest attainable standard of health is one of the fundamental rights of all human beings without distinction as to race, colour and religion’.

Articles 12, 16 and 21–23 in Uganda's Constitution provide for free access to health care, emergency care and the related patients’ rights which stipulate that ‘the State shall take all practical measures to ensure the provision of basic medical services to the population, provide for equality amongst all people and guarantee a healthy State’. Similarly, the Bill of Rights in Uganda's Constitution, the health professional Acts^9, 10, 11^ and Code of Conduct and Ethics for Uganda Public service^12^ provide for autonomy, beneficence, justice, informed consent and non-malficience as basic human rights. *The Uganda Medical and Dental Practitioners Act*,^9^ chapter 272, provides for the ‘right to treatment based on clinical need regardless of one's ability to pay; participation in health care decisions; accessing information about HW providing care, patients’ diseases and treatment; respect, dignity and privacy’.^13, 14^ Thus the Uganda Patients’ Charter, 2009 is a public health policy for creating awareness of and promoting the practice of patients’ rights at public facilities in Uganda.

Despite the constitutional provision for patients’ rights, Twinomugisha^15^ and the Uganda National Health Users'or Consumers’ Organisation (UNHCO)^16^ report that ‘Uganda's public health system continues to inadequately promote and protect its patients’. The WHO also cautions that ‘the existence of Patients’ Charters without efforts to raise awareness amongst patients does not improve the quality of health care’.^13^ UNHCO reports that patients accessing health care face unethical practices such as neglect of duties by health workers (HWs) and non-provision of a minimum health package,^17^ and are thus denied their rights to confidentiality and timely access to health services.^14, 18, 19^

In Uganda poor and vulnerable patients, who include the mentally ill, those with HIV and AIDS, lesbian, gay, bisexual, transgender and intersexual patients, and women, are at risk of abuse of their rights.^20^ Various reports have related the abuse of the rights to limited resources for health care,^16, 21, 22, 23^ such as is the case in Uganda where only 56% of health posts are filled and there is only one doctor for every 22 000 patients.^21^

UNHCO reports that patients who are inquisitive about their rights are often misinterpreted by HWs as doubting their clinical skills, and are perceived to want to know more than they deserve and as unappreciative,^16^ contrary to provisions in the Code of Conduct and Ethics for Uganda Public Service.^12^ Despite efforts by UNHCO and the Ministry of Health (MoH) quality assurance department to raise awareness and promote integration of patients’ rights in care at Mulago Hospital, reports on patient strikes and dissatisfaction remain common. The extent to which the Uganda Patients’ Charter has been operationalised since its launch in 2009 remains largely unknown.

## Significance

This study offers opportunities for identifying strategies whereby policy, legislative and programmatic developments can strengthen systems for practice of patients’ rights in resource constrained public health settings towards improving health outcomes.

## Objective

The study aimed at assessing the level of awareness, responsiveness and practice of patients’ rights amongst patients and HWs at Mulago national referral hospital in Uganda.

## Methods

In this study awareness refers to the knowledge of and ability to recognise the patients’ rights; responsiveness refers to strategies or systems put in place to promote, address and respond to patients’ rights issues; and practice refers to behaviours regarding patients’ rights during a HW-patient health interaction.

### Study design and setting

The study adopted a three-phase cross-sectional questionnaire-based descriptive survey design amongst patients, HWs and key informants at Uganda's Mulago national referral hospital in the capital city of Kampala. Mulago serves as a teaching hospital with a 1500-bed capacity and an annual inpatient and outpatient turnover in excess of 140 000 and 600 000 respectively. The hospital provides a wide range of health care and research services through various health professionals and trainees to diverse patient populations in five main clinical departments.

### Data collection process

A team of five trained and experienced research assistants together with the principal investigator collected qualitative and quantitative data using pretested and standardised questionnaires during the study period May–June 2012. The questionnaires specifically addressed aspects of awareness of, responsiveness to and practice of 43 patients’ rights as outlined in the Uganda Patients’ Charter (2009). The proportion of HWs and patients who were knowledgeable on each of the 43 specific patients’ rights in the Charter was calculated. The level of awareness was then determined on a scale of high (> 80%), moderate (70-79%), fair (60-69%) and poor (< 60%).

For patients who could not speak English the interviewer-administered questionnaires were translated into local languages, including Luganda and Luo. The patient and HW respondents were recruited from the five departments of the hospital: surgical, medical, paediatrics, general nursing and therapeutics. Three target populations were considered in this study: all patients receiving health care at Mulago Hospital; all HWs at the facility, which included professionals, interns and students providing care; and clinical heads of departments (HoD) and administrators who served as key informants (KI).

### Sampling and sample frame

At each of the five departments of the hospital at least half of the existing clinics were purposively selected for inclusion in this study based on daily patient load and involvement with patients. A total of 28 clinics were purposively selected for this study, from which patients and HWs were recruited. To prevent a change in behaviour amongst HWs regarding the practice of patients’ rights, data were collected in three phases: patient interviewer-administered questionnaire exit interview; HW self-administered questionnaires; and KI interviews.

The KI included 10 HoD and administrators at the hospital and 6 at the Uganda MoH quality assurance department, civil society organisations (CSOs) and health professional councils. These KI, who are stakeholders in the implementation of patients’ rights in Uganda, provided qualitative data on extent of and factors influencing awareness, responsiveness and practice of patients’ rights at the facility.

A total of 211 patients, 98 HW and 16 KI participated in this study. Patient and HW sample sizes were determined using the Leslie Kish (1965)^24^ method, and the KI were purposively selected by virtue of their responsibility. A proportionate sampling method was used to determine the number of patients to be interviewed at each department and clinic, according to the estimated daily patient attendance records of the hospital, department and clinic.

The number of outpatients and inpatients to be interviewed at target departments and clinics was estimated in a ratio of 40:60 from a preliminary survey prior to the study based on attendance registers at the target clinics. Simple random sampling of patients to be interviewed at each clinic was performed using inpatient and outpatient registers as the sampling frames. A total of 98 HWs was randomly selected based on the duty roster for each department. Ten clinical HoD and administrators at the hospital and 6 administrators at the MoH, CSOs and health professional councils were purposively selected by virtue of their role in patients’ rights issues.

### Selection criteria

The study included all patients aged 18 years and above receiving care services at Mulago Hospital in the study period who gave informed consent; guardians of patients below the age of 18 years who gave informed consent to participate in the study; and guardians of very ill patients. A ballot system was used to select names of HWs under a particular cadre as they appeared on the HW register of each department. Only HWs who were on duty at the time of the study and gave informed consent were included. The HWs in this study included doctors, nurses, clinical officers, consultants, laboratory technologists, pharmacists, anaesthetists and orthopaedic officers. Key informants were clinical heads of the five selected departments in Mulago Hospital. A snowball method was used to identify other administrators within the hospital with knowledge or responsibility related to awareness of, responsiveness to and practice of patients’ rights.

### Data analysis

Quantitative data were double entered into Epidata v3.1 software and exported to the Statistical Package for Social Sciences version 20 for quantitative analysis. The qualitative data were manually coded and thematically analysed. Main outcome variables were social demographic characteristics, level of awareness, responsiveness, practice of patient rights and associated factors.

### Ethical considerations

The study was approved by the Institutional Research and Ethics boards of Makerere University School of Public health and Mulago Hospital. All study respondents gave informed consent prior to participating in the study and all interviews were conducted on the basis of anonymity.

## Results

### Demographic characteristics

A total of 211 patients were interviewed. Most of the patients were female (63%); outpatients (60%); in the age category of 21-40 years (69%); had attained at least secondary education (72%); were married (55.5%); not in formal employment (83.6%); and resided in central Kampala (53.1%) (Table 1).

**TABLE 1:** Demographic profiles of patients and awareness levels in the study (*N* = 211).

Characteristic	% patients	% aware (know patients’ rights)	*p* value
**Sex**			
Female	63	26.2	0.192
Male	37	18.6	-
**Age (years)**			
< 20	12	3.8	0.387
21-40	69	33.0	-
41-60	16	7.2	Mean age
61-80	1	0	(32.7 ± 11.7)
> 80	1	0.5	
**Level of education**			
Tertiary	26	21.0	-
Secondary	46	17.6	0.000*
Primary	22.7	5.2	-
No education	7.6	1.0	-
**Marital status**			
Married	55.5	25.5	-
Single	36	18.3	0.074
Divorced/widowed	8.5	1.0	-
**Residence by region**			
Central Kampala	53.1	28.5	0.096
Central - other	20.9	7.2	-
Western	7.6	1.9	-
Eastern	10	4.3	-
Northern	6.6	2.4	-
**Employment status**			
Self-employed	42.7	18.6	0.000*
Unemployed	34.1	9.0	-
Formal employment	16.6	14.3	-
Casual labourer	6.6	2.9	-
**Patient visit status**			
New patient	28	9.6	0.048*
Revisit patient	72	35.1	-
**Type of visit**			
Outpatient	58.8	24.3	0.253
Inpatient	41.2	20.5	-

* *p* value significant, as determined by Pearson Chi-square one sample test at the 95% confidence interval.

Amongst the 98 participating HWs the age range was 20–62 years; there were similar numbers of male (49%) and female (51%) HWs, two-thirds had at least a degree qualification and 61% were married (Table 2). Of the 16 KI, 10 (63%) were HoD or administrators at the hospital and 6 (37%) were stakeholders regarding patients’ rights-related issues based at the MoH, CSO and health professional councils. Most of the KI (62.5%) had been in positions of leadership for more than 3 years, with a mean duration in the position of 8.6 years and a range of 3 months to 19 years.

**TABLE 2:** Demographic profiles of health workers and awareness of rights (*N* = 98).

Characteristic	% HWs	% Aware (ever heard/read about patients’ rights	*p* value
**Sex**			
Female	49	44.9	0.374
Male	51	43.9	-
**Age (in years)**			
20-30	45	36.7	0.219
31-40	36	34.7	median age:
41-50	12	12.0	(31.50 ± 8.5)
51-60	6	5.1	-
**Level of education**			
PhD	2	2	-
Masters	15	14	-
Postgraduate diploma	2	1	0.000*
Degree	39	33	-
Diploma	38	32	-
Certificate	4	4	-
**Length of service (years)**			
0-5	43	40	-
6-10	23	21	0.000*
11-15	12	9	-
16-20	8	8	-
> 20	13	9	-

* *p* value significant as determined by Pearson Chi-square one sample test at the 95% confidence interval.

### Awareness of patients’ rights amongst patients and HWs

Most patients (81.5%) and HWs (69.4%) had never heard of the Uganda Patients’ Charter. Over half of the patients (55.5%) indicated that they did not fully know their rights as patients, despite 72% having a secondary education. Regarding the specific rights in the Charter, 60% of the patients expressed knowledge of at least half or 25 (58%) of the 43 rights. However, most patients (> 80%) knew of only 10 of the 43 rights (23.3%), compared to > 80% of HWs who knew of up to 30 (69.8%) of the 43 specific patients’ rights (Table 3).

**TABLE 3:** Awareness of patients’ rights amongst patients and HW.

Scale by% of respondents	Number and percentage of patients’ rights which respondents knew (*n* = 43 rights)	
	
	Patient awareness	HW awareness
Highly knowledgeable (80-100%)	10 (23.3%)	30 (69.8%)
Knowledgeable (70-79%)	7 (16.3%)	6 (13.9%)
Fairly knowledgeable (60-69%)	8 (18.6%)	2 (4.7%)
Poorly knowledgeable (< 60%)	18 (41.8%)	5 (11.6%)

### Responsiveness

Patients’ rights-related information was displayed on very few department or clinic notice boards at Mulago Hospital. Patients had access to information at an information centre outside of the hospital which was remote to most of them. Posters on patients’ rights were displayed on the hospital's administration notice board. KI and HWs at the hospital reported participating in community and media sensitisation campaigns and continuing medical education to create awareness of patients’ rights. KI at the hospital reportedly supervise HWs at points of care to ensure practice of patients’ rights.

Only 46% of HWs reported engaging in activities to create awareness of patients’ rights during patient consultations or community campaigns. Aspects commonly communicated to patients included nature of disease and treatment, health services offered, consent, procedures to be undertaken, arriving at a decision and implications.

### Practice of patients’ rights

None of the HWs practised all of the 43 patients’ rights as stated in the Uganda Patients’ Charter. Most HWs (80%) practised at least two-thirds of the 43 patients’ rights. Over 50% of patients reported HWs having ensured that their rights were exercised whilst accessing services at clinics in the hospital. At least 36.5% of the patients reportedly faced at least one challenge to experiencing their rights during contact with Mulago Hospital. Most (79%) of the patients whose rights were violated at the hospital did not request their rights. Patients identified discrimination based on their ability to bribe the HW with a tip, their socio-economic class, political status and affiliation, prior knowledge and relationship to the HW, and tribal favouritism by HWs as impediments to accessing their rights (Table 4).

**TABLE 4:** The most practised rights as indicated by patients and HWs.

Aspect of specific patients’ rights; the right to:	Reported practice (%)	
	
	Patients (*N* = 211)	HWs (*N* = 98)
Confidentiality and privacy	200 (94.8)	85 (87)
Medical care	190 (90.1)	85 (87)
Healthy and safe environment	187 (88.6)	78 (80)
Non-discrimination: No discrimination against patients receiving care	173 (82.0)	73 (74)
Proper medical care with regard to professionalism and quality assurance	178 (84.3)	81 (83)
Safety and security with regard to hospital environment	182 (86.2)	79 (81)
Informed consent	155 (73.5)	75 (77)
Continuity of care	139 (65.9)	66 (67)
1. Right to confidentiality and privacy	174 (82.4)	61 (62)
1.1 Refusal of treatment	208 (98.6)	61 (62)
Participation in decision making on treatment	64 (30.3)	24 (24)
2. Receiving visitors	46.4 of the 100 (47.4%)	84 (86)
3. Medical care without consent (Percentage patients never given medical care without consent)	171 (81)	54 (55)
Percentage patients ever participated in research / training who consented to voluntary participation/had consenting process witnessed	43 (20.3)	50 (51)
	39 (18.4)	46 (47)
	35 (16.6)	36 (37)
**The least practiced patient's rights**		
Right to redress	79 (37.5)	42 (43)
Right to confidentiality	99 (46.9)	52 (53)
Participation in decision making	74 (35.1)	79 (81)
Participation or representation in development of health policies	28 (13.3)	58 (59)
Treatment by a named HW: HW's name always revealed before asking	74 (35.0)	57 (58)
Patients ever asked for a copy of their medical information	38 (18)	72 (73)

The right to confidentiality and privacy, medical care, a healthy and safe environment and safety and security are those most practised at Mulago Hospital. The right to redress, participation in decision making and treatment by a named HW were those least practised.

### Factors perceived to influence awareness and practice of patients’ rights

Thematic analysis of qualitative data showed that patients’ illiteracy, language barriers, low socio-economic status, feeling inferior to HWs and perceived favour to access free services were perceived by patients as hindering negotiating power for their rights. HWs perceived fear of litigation, non-cooperation from some patients, high workload-related stress and HWs’ superiority complexes as hindering exposure and empowerment of patients.

KI reported that lack of good work ethics by HWs, such as neglect of duties and non-patient-focused care, exposes patients to discrimination. Institution-related factors such limited human and financial resources, unclear communication channels to access patient rights information and seek redress, and poor social and customer care skills hamper practice of patients’ rights at the hospital.

**FIGURE 1:**
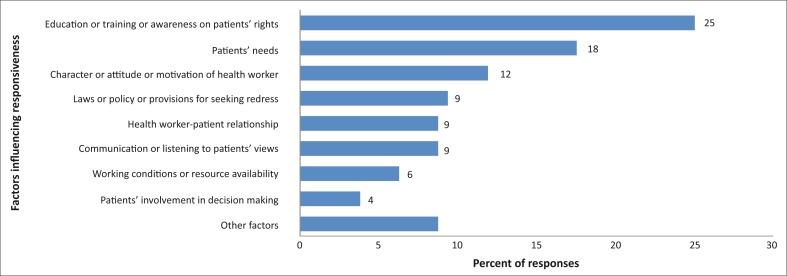
Factors perceived to influence responsiveness (*n* = 160 responses). ‘Other factors’ listed by HWs include politics, corruption and greed; and negligence of leaders, especially on monitoring, which results in reluctance of HWs to implement patients’ rights. Empathy, staff remuneration, poverty and the existing code of conduct and ethics of HWs were also mentioned as factors influencing responsiveness to patients’ rights.

Prior training on patients’ rights, patients’ health needs, HWs’ attitude towards work, handling vulnerable patient populations, HWs’ empathy and laws and policies such as constitutional rights and professional acts, as well as the existence of mechanisms for redress influence response to rights, as indicated in Figure 1.

## Discussion

This study shows that despite provisions for the practice of patients’ rights in the Bill of Rights in Uganda's Constitution, the *Medical and Dental Act*, *Pharmacy Act*, *Allied Professionals Act* and Uganda Patients’ Charter of 2009, awareness of, responsiveness to and practice of patients’ rights remain limited at Mulago Hospital. Patients encounter challenges in accessing their rights.

Similar studies have reported that despite the existence of laws, Uganda's public health system inadequately promotes and protects and frequently violates the rights of patients seeking care.^15, 16, 17^ UNHCO^16^ and Twinomugisha^15^ showed similar findings of violation of patients’ rights prior to the launch of the Charter. Human Rights Network Uganda^25^ attributes this to the poor socio-economic status of patients who are vulnerable to abuse and resource constraints to meet the health care needs of public hospitals due to poor funding, poor remuneration of HWs and high patient turnover. These factors facilitate unethical behaviour and patient exploitation, such as charging illegal fees and denying patients their right to health.

Unfortunately systems of checks and balances on the practice of patients’ rights at the hospital remain unclear to both patients and HWs, contrary to provisions in the Constitution that the State will take all efforts to ensure provision of health services to its population.

A similar study by Ducinskiene^18^ in Lithuania showed limited awareness amongst patients (56%). Surveys by UNHCO^16^ and the Action Group for Health, Human Rights and HIV/AIDS^19^ in Uganda showed that awareness of patients’ rights issues amongst patients (50%) and HWs remained poor. Inadequate awareness resulting from limited availability of requisite information has been shown to limit the capacity of Ugandans to demand their health rights, and that this translates into poor health outcomes.^7^

The current responsive strategies to the Uganda Patients’ Charter through use of posters on administrative notice boards remains limited and remote from points of care; the information centre lies outside of the hospital and is inaccessible to many patients and HWs. HWs rarely engage in community-based patients’ rights activities.

Such strategies have been found to be largely ineffective as many patients and HWs were not aware of their existence. There is a lack of a clear system for patients to seek redress and guide access to their rights. The system is perceived as reactive rather than proactive and only comes into place when patients’ rights have been violated. Responsiveness has been strained by the slim budget on which the hospital operates and thus largely depends on CSOs such as UNHCO to create awareness, a mechanism that may not be sustainable. There is therefore a need for the ownership and integration of a patient's rights approach in patient care.

Patients remain largely passive as they do not feel adequately empowered or prepared to demand their rights in a health care system which they perceive as doing them a favour, and which lacks systems that clearly address their rights. Patients being less informed influences their health care-seeking behaviours.^16, 26^

The practice of patients’ rights at the hospital is driven by HWs’ empathy towards a patient's needs rather than full awareness of the rights concept. Existing mechanisms to promote patients’ rights at the hospital largely focus on patients’ needs and only sparsely emphasise the role of the HWs and health care system. High patient turnover and lack of time and resources means most HWs are not motivated to engage in sensitisation of communities and patients on patients’ rights.^16, 23, 27^ This is unethical and unlawful as per the professional codes of conduct, where HWs are required to disclose information to and educate patients on their rights.

The study shows that although the Uganda Patients’ Charter^3^ entitles patients to high-quality services in public hospitals, unethical conduct such as charging illegal fees, discrimination, absconding from duty, negligence, rudeness to patients and physical and emotional abuse deter timely access to and practising of patients’ rights.^2, 19^ It shows that intrinsic patient, HW and health system factors impede the awareness and practice of patients’ rights. Implementation of the Charter needs to take into account all of the confounding factors, and to maximise the limited human and budget resources which impact on service delivery and awareness of, responsiveness to and practice of patients’ rights.^17, 21, 23^

Limited resources at the hospital don't fully justify the denial of simple practices such as the HWs revealing their names and professional identity, which aids accountability and builds trust in the HW and health system and provides a reference point for continuity in care and seeking redress. A similar study by the National Institute for Patients Rights^28^ reported that most patients who are abused and HWs who provide care never provide their identity.

## Conclusion and recommendations

Awareness of and access to patients’ rights and related information, including the Uganda Patients’ Charter, remain limited and remote for patients seeking care at Mulago Hospital. There is a need to strengthen the existing patients’ rights awareness strategies through policies that promote a patients’ rights-based care approach at the points of care. There is a need for the integration of social services in patient care to create awareness of rights amongst patients. National awareness campaigns on patient rights that adopt a multilevel, multichannel approach to increase awareness amongst patients and HWs are urgent.

Patients believe that access to health care at Mulago Hospital is a favour rather than a right, and thus rarely demand their rights for fear of being discriminated against whilst accessing care. The need for multiple literacy and linguistic approaches, including audiovisual messages at points of care in the context of the illiterate, anxious and sick patient population is urgent.

An opportunity for a patient-HW forum may foster better understanding of each one's responsibility. The hospital should establish a grievance committee or designated person to oversee implementation of patients’ rights and act as a point of redress.

Violation of patients’ rights, discrimination and unethical practices during patient care remain common at Mulago Hospital. There is a need for strategies aimed at enforcing ethical conduct in the public service and creating an independent system for seeking redress. There is also a need to evaluate the effectiveness of the current strategies for promoting patients’ rights, in order to understand the intrinsic factors that drive the demand for rights and responsiveness in the context of a resource-limited setting.

The MoH should establish and extensively publicise guidelines for patients to report cases of violation of their rights. The MoH should also enhance legal education of HWs and citizens, and provide legal assistance to abused patients. Inadequate resources for health promote unethical practices such as HWs demanding illegal fees and thus propagating the abuse of patients’ rights. The MoH should commit more funds to ensure availability of basic resources for implementing patients’ rights issues, and improve supervision of HWs.
